# The relationship between nut intake and risk of colorectal cancer: a case control study

**DOI:** 10.1186/s12937-018-0345-y

**Published:** 2018-03-07

**Authors:** Jeeyoo Lee, Aesun Shin, Jae Hwan Oh, Jeongseon Kim

**Affiliations:** 1 Department of Preventive Medicine, Seoul National University College of Medicine, 103 Daehakro, Jongno-gu, Seoul, 03080 South Korea; 2 Cancer Research Institute, Seoul National University, 103 Daehakro, Jongno-gu, Seoul, 03080 South Korea; 3 Center for Colorectal Cancer, National Cancer Center, 323 Ilsan-ro, Ilsandong-gu, Goyang-si, 10408 Gyeonggi-do South Korea; 4 Department of Cancer Biomedical Science, Graduate School of Cancer Science and Policy, National Cancer Center, 323 Ilsan-ro, Ilsandong-gu, Goyang-si, 10408 Gyeonggi-do South Korea

**Keywords:** Nut, Colorectal cancer, Case-control study, Korea

## Abstract

**Background:**

Nut consumption is known to reduce the risk of obesity, diabetes mellitus, and cardiovascular disease. However, in previous studies, portion sizes and categories of nut consumption have varied, and few studies have assessed the association between colorectal cancer risk and nut consumption. In this study, we investigated the relationship between nut consumption and colorectal cancer risk.

**Methods:**

A case-control study was conducted among 923 colorectal cancer patients and 1846 controls recruited from the National Cancer Center in Korea. Information on dietary intake was collected using a semi-quantitative food frequency questionnaire with 106 items, including peanuts, pine nuts, and almonds (as 1 food item). Nut consumption was categorized as none, < 1 serving per week, 1–3 servings per week, and ≥3 servings per week. A binary logistic regression model was used to estimate odds ratios (OR) and their 95% confidence intervals (CI) for the association between nut consumption and colorectal cancer risk, and a polytomous logistic regression model was used for sub-site analyses.

**Results:**

High nut consumption was strongly associated with reduced risk of colorectal cancer among women (adjusted ORs: 0.30, 95%CI: 0.15–0.60 for the ≥3 servings per week group vs. none). A similar inverse association was observed for men (adjusted ORs: 0.28, 95% CI: 0.17–0.47). In sub-site analyses, adjusted ORs (95% CIs) comparing the ≥3 servings per week group vs none were 0.25 (0.09–0.70) for proximal colon cancer, 0.39 (0.19–0.80) for distal colon cancer, and 0.23 (0.12–0.46) for rectal cancer among men. An inverse association was also found among women for distal colon cancer (OR: 0.13, 95% CI: 0.04–0.48) and rectal cancer (OR: 0.40, 95% CI: 0.17–0.95).

**Conclusions:**

We found a statistically significant association between high frequency of nut consumption and reduced risk of colorectal cancer. This association was observed for all sub-sites of the colon and rectum among both men and women, with the exception of proximal colon cancer for women.

## Background

Total global consumption of nuts has increased 59% over the past decade [1]. Per capita consumption of seeds and nuts in the Korean population was 3.0 g/day in 1998, which increased to 7.6 g/day in 2015 [2]. The US Food and Drug Administration approved the labelling of food products containing nuts to indicate that nuts may reduce the risk of heart-related diseases.

Nuts contain many nutrients, including high quality vegetable protein, fat, unsaturated fatty acids, fiber, vitamins (e.g., vitamin E, vitamin B_6_ folate, niacin), minerals (e.g., zinc, potassium, calcium, magnesium), phytochemicals (e.g., flavonoid, carotenoids, phytosterols), and other bioactive compounds [3–8]. These nutrients may reduce the risk of overall mortality [9, 10] and incidence of colorectal and endometrial cancer [11], cardiovascular diseases [12], type 2 diabetes [13, 14], and metabolic syndrome [15]. These health effects may be due to various mechanisms, including antioxidant activity [16], reduction of DNA damage [17], regulation of inflammatory response and immunological activity [18], and anti-carcinogenic effects [19].

Few epidemiologic studies have assessed the association between nut consumption and risk of colorectal cancer. Both the Nurses’ Health Study [20] and the Adventist Health Study [21] conducted in the United States suggested a null association. In the European Prospective Investigation into Cancer and Nutrition study [22], women in the highest nut and seed intake group had a significantly decreased risk of colon cancer. The results of studies examining peanut consumption and colorectal cancer risk in Taiwan [23] have suggested an inverse association for women. Several previous case-control studies have also been implemented, but results were conflicting [24–26].

The type and recipe of Korean nut intake differs from that in other countries. Nuts are mainly consumed as snacks in Korea [27]. Previous studies have grouped nuts along with peanut butter and seeds [20, 22], or had only one category for peanut products and did not consider tree nuts or seeds [23]. In addition, few studies have assessed the association of nut consumption with colorectal cancer by sub-site [20, 22]. In the current study, we investigate the relationship between nut intake and colorectal cancer risk by sub-site among Korean adults.

## Methods

### Study population

Data on newly diagnosed colorectal cancer cases were collected from August 2010 to August 2013 at the Center for Colorectal Cancer of the National Cancer Center in Korea. To minimize recall bias, colorectal cancer patients were invited to participate in the study while they were hospitalized for cancer diagnosis or surgery. Of 1427 eligible cases, 1259 were invited, and 1070 agreed to participate. A total of 168 patients were excluded for the following reasons: 1) difficulty hearing or communicating or 2) unavailable to meet in person during their hospital stay. We also excluded cases with no record of completing a semi-quantitative food frequency questionnaire (SQFFQ) (145 cases) and cases who reported energy intakes of less than 500 kcal/day or greater than 4000 kcal/day (2 cases). Controls were selected from among the Korean population who had health screenings through the National Health Insurance program at the same hospital where the cases were treated. From among the 14,201 visitors at the hospital between October 2007 and December 2014, participants with an incomplete SQFFQ (*n* = 5044) and 120 participants who reported inadequate or excessive intake of calories (< 500 kcal/day or ≥ 4000 kcal/day) were excluded. Cases and qualified controls (*n* = 9037) were matched on sex and 5-year age group (1:2 ratio). Consequently, the final analysis included 923 cases and 1846 controls. All participants were given a detailed description of the study, and written informed consent was collected from all participants. The present study guidelines were approved by the Institutional Review Board of the National Cancer Center (IRB No. NCCNCS-10-350 and NCC 2015–0202).

### Data collection

Data on general characteristics; family history of cancer; drinking, smoking, and exercise habits; and dietary intakes were collected by a trained dietitian using structured questionnaires. Dietary information was examined using an SQFFQ that was developed by the Korea Centers for Disease Control and Prevention and whose reliability and validity have been demonstrated [27]. The SQFFQ was developed based on the Korean National Health and Nutrition Examination Survey, which was conducted in 1998. Food items were selected based on the cumulative percentage contribution and cumulative multiple regression coefficients of 17 major nutrients. The SQFFQ was designed to measure typical food intake habits during the course of one year and comprised 106 food items, including peanuts, pine nuts, and almonds (as 1 food item). Data from completed SQFFQs were used to calculate daily nut and calorie intake by using the Nutritional Analysis Program for Professionals, ver. 4.0 (CAN-Pro 4.0 the Korean Nutrition Society, 2012, Seoul, Korea). The SQFFQ had 9 levels of frequency (‘none or little’, ‘once a month’, ‘2–3 times a month’, ‘1–2 times a week’, ‘3–4 times a week’, ‘5–6 times a week’, ‘once a day’, twice a day’ and ‘3 times a day’) and 3 categories for portion size (1/2 serving, 1 serving, 1–1/2 servings); 1 serving was considered to be 15 g. Average nut consumption for each type of nut was categorized as none, < 1 serving per week, 1–3 servings per week, and ≥3 servings per week. Detailed clinical information on colorectal cancer was obtained from medical records and sub-sites were classified into three categories: proximal colon (cecum, ascending colon, hepatic flexure, transverse colon, and splenic flexure), distal colon (descending colon, sigmoid-descending colon junction, sigmoid colon) and rectum (rectosigmoid colon, rectum) [28].

### Covariates

Based on the literature, the following potential confounding variables were considered in the analyses [9, 11, 13, 20, 22, 29]: age (age < 50 years, age 50–59, and age 60 or older), education level (less than high school, high school, college or above), body mass index (BMI; < 25 kg/m^2^, ≥25 kg/m^2^), alcohol consumption (non-drinker, ex-drinker, current drinker), regular exercise (no, yes). We also considered dietary factors including intakes of fruits and vegetables, red meat, calcium, and vitamin D, as well as total energy intake (continuous). Missing data for categorical covariates were included in the multivariate logistic regression models as a dummy category.

### Statistical analysis

Participant characteristics were compared by using Pearson’s Chi-square tests for categorical variables and general linear regression for continuous variables. Considering multi-collinearity, the final model included age; education level; alcohol consumption; BMI; regular exercise; intakes of red meat, fruits and vegetables, calcium, and vitamin D; and total energy intake by using residual methods. Binary logistic regression models were used to estimate odds ratios (OR) and their 95% confidence intervals (CI) for associations between nut consumption and colorectal cancer risk. Subgroup analyses of nut consumption and colorectal cancer risk by cancer sub-site (anatomical locations) were conducted using polytomous logistic regression models. All statistical analyses were stratified by sex and performed using SAS software (version 9.4; SAS Institute Inc. Cary, NC, USA).

## Results

Table 1 presents the basic characteristics and demographics of colorectal cancer cases and matched controls. Among the 923 colorectal cancer cases, five cases were hereditary nonpolyposis colorectal cancer and three cases were familial adenomatous polyposis. In both sexes, colorectal cancer cases tended to have lower education levels, have lower household incomes, not be engaged in regular exercise, have higher total energy intake, be a current smoker, or have a first-degree family history of colorectal cancer compared with controls. Red meat and fruit and vegetable intakes were lower among cases compared with controls.

**Table 1:** General characteristics of cases and controls stratified by sex, N (%)

	Men (*n* = 1875)			Women (*n* = 894)		
Variable	Cases (*n* = 625)	Controls (*n* = 1250)	*P*-value^a^	Cases (*n* = 298)	Controls (*n* = 596)	*P*-value^a^
Age (years)^b^	57.2 ± 9.4	56.6 ± 8.7	0.173	55.3 ± 10.2	54.9 ± 9.8	0.592
Marital status			<.001			<.001
Married	557(89.1)	1162(93.0)		216(72.5)	493(83.7)	
Single	66(10.6)	72(5.8)		80(26.9)	98(16.5)	
Missing	2(0.3)	16(1.2)		2(0.5)	5(0.8)	
Educational level			<.001			<.001
Less than high school	183(29.2)	175(14.0)		138(46.3)	106(17.8)	
High school	266(42.6)	329(26.3)		103(34.6)	258(43.3)	
College or above	176(28.2)	712(57.0)		57(19.1)	223(37.4)	
Missing	0(0.0)	34(2.7)		0(0.0)	9(1.5)	
Income (1000 won/month)			<.001			<.001
< 2000	222(35.5)	254(20.3)		99(33.2)	134(22.5)	
2000–4000	253(40.5)	534(42.7)		134(45.0)	218(36.6)	
> 4000	150(24.0)	363(29.1)		65(21.8)	184(30.9)	
Missing	0(0.0)	99(7.9)		0(0.0)	60(10.0)	
Body mass index (kg/m^2^)			<.001			0.270
< 25	432(69.1)	734(58.7)		207(69.5)	435(73.0)	
≥ 25	192(30.7)	516(41.3)		91(30.5)	161(27.0)	
Missing	1(0.2)	0(0.0)		0(0.0)	0(0.0)	
Smoking status			0.076			<.001
Non-smoker	145(23.2)	245(19.6)		264(88.6)	571(95.8)	
Ex-smoker	303(48.5)	671(53.7)		15(5.0)	16(2.7)	
Current smoker	177(28.3)	334(26.7)		19(6.4)	9(1.5)	
Missing	0(0.0)	0(0.0)		0(0.0)	0(0.0)	
Alcohol consumption			0.001			0.187
Non-drinker	107(17.1)	199(16.0)		172(57.7)	362(60.7)	
Ex-drinker	103(16.5)	136(10.9)		26(8.7)	33(5.5)	
Current drinker	415(66.4)	915(73.1)		100(33.6)	201(33.7)	
Missing	0(0.0)	0(0.0)		0(0.0)	0(0.0)	
Regular exercise			<.001			<.001
No	387(61.9)	490(39.2)		225(75.5)	262(44.0)	
Yes	238(38.1)	715(57.2)		73(24.5)	333(55.9)	
Missing	0(0.0)	45(3.6)		0(0.0)	1(0.1)	
First-degree family history of cancer			0.002			0.141
No	392(62.7)	686(54.9)		171(57.4)	311(52.2)	
Yes	233(37.3)	560(44.8)		127(42.6)	285(47.8)	
Missing	0(0.0)	4(0.3)		0(0.0)	0(0.0)	
First-degree family history of colorectal cancer			<.001			0.926
No	560(89.6)	1188(95.0)		277(93.0)	555(93.1)	
Yes	65(10.4)	58(4.6)		21(7.1)	41(6.9)	
Missing	0(0.0)	4(0.4)		0(0.0)	0(0.0)	
Fruit and vegetable intake g/d^b^	279.4 ± 155.7	350.2 ± 236.7	<.001	343.1 ± 192.8	470.7 ± 383.4	<.001
Red meat intake, g/d^b^	56.0 ± 36.2	64.4 ± 41.9	<.001	40.9 ± 26.9	43.7 ± 28.7	<.001
Energy intake, kcal/d^b^	2127.7 ± 509.1	1731.6 ± 545.8	<.001	1814.4 ± 523.5	1604.6 ± 577.4	<.001

^a^*P*-values were calculated by using the chi-square test for categorical variables and linear regression for continues variables. ^b^ Mean (s.d.) ^c^ Fruit and vegetable intake and red meat intake were adjusted for total individual energy intake by using the residual method

Table 2 describes the general characteristics of the study participants according to nut consumption. Men and women with higher frequencies of nut consumption tended to have higher levels of education, more regular exercise, higher mean fruit and vegetable intake, and higher total energy intake.

**Table 2:** General characteristics of study participants by nut consumption frequency, N (%)

	Men (n = 1875)					Women (n = 894)				
Variable	None	< 1 serving per week	1–3 servings per week	≥3 servings per week	*P*-value^a^	None	< 1 serving per week	1–3 servings per week	≥3 servings per week	*P*-value^a^
Number	682	855	163	175		280	413	85	116	
Age (years) ^b^	56.1 ± 9.4	57.0 ± 8.8	57.4 ± 8.2	58.0 ± 8.3	0.032	54.4 ± 11.0	55.4 ± 9.6	53.1 ± 9.0	56.5 ± 8.8	0.060
Marital status					0.706					0.028
Married	622(91.2)	790(92.4)	149(91.4)	158(90.2)		212(75.7)	323(78.2)	76(89.4)	98(84.5)	
Single	56(8.2)	57(6.7)	13(8.0)	12(6.9)		65(23.2)	87(21.1)	9(10.6)	17(15.7)	
Missing	4(0.6)	8(0.9)	1(0.6)	5(2.9)		3(1.1)	3(0.7)	0(0.0)	1(0.8)	
Education level					0.001					<.001
Less than high school	149(21.9)	156(18.2)	23(14.1)	30(17.1)		104(37.1)	113(27.4)	9(10.6)	18(15.5)	
High school	241(35.3)	268(31.4)	42(25.8)	44(25.1)		121(43.2)	159(38.5)	36(43.4)	45(38.8)	
College or above	282(41.4)	419(49.0)	92(56.4)	95(54.3)		53(18.9)	136(32.9)	39(45.9)	52(44.8)	
Missing	10(1.4)	12(1.4)	6(3.7)	6(3.5)		2(0.8)	5(1.2)	1(1.1)	1(0.9)	
Income (1000 won/month)					0.532					0.066
< 2000	182(26.7)	223(26.1)	35(21.5)	36(20.6)		81(28.9)	113(27.4)	19(22.4)	20(17.2)	
2000–4000	284(41.6)	363(42.5)	67(41.1)	73(41.7)		115(41.1)	159(38.5)	33(38.8)	45(38.8)	
> 4000	178(26.1)	231(27.0)	51(31.3)	53(30.3)		63(22.5)	118(28.6)	25(29.4)	43(37.1)	
Missing	38(5.6)	38(4.4)	10(6.1)	13(7.4)		21(7.5)	23(5.5)	8(9.4)	8(6.9)	
Body mass index (kg/m^2^)					0.294					0.852
< 25	434(63.6)	520(60.8)	109(66.9)	103(58.9)		199(71.1)	294(71.2)	62(72.9)	87(75.0)	
≥ 25	247(36.2)	335(39.2)	54(33.1)	72(41.1)		81(28.9)	119(28.8)	23(27.1)	29(25.0)	
Missing	1(0.2)	0(0.0)	0(0.0)	0(0.0)		0(0.0)	0(0.0)	0(0.0)	0(0.0)	
Smoking status					0.207					0.194
Non-smoker	152(22.3)	167(19.5)	35(21.5)	36(20.6)		257(91.8)	385(93.2)	82(96.5)	111(95.7)	
Ex-smoker	329(48.2)	456(53.3)	88(54.0)	101(57.7)		9(3.2)	15(3.6)	2(2.4)	5(4.3)	
Current smoker	201(29.5)	232(27.2)	40(24.5)	38(21.7)		14(5.0)	13(3.2)	1(1.1)	0(0.0)	
Alcohol consumption					0.913					0.492
Non-drinker	117(17.2)	135(15.8)	24(14.7)	30(17.1)		161(57.5)	253(61.2)	51(60.0)	69(59.5)	
Ex-drinker	80(11.7)	117(13.7)	20(12.3)	22(12.6)		17(6.1)	30(7.3)	8(9.4)	4(3.4)	
Current drinker	485(71.1)	603(70.5)	119(73.0)	123(70.3)		102(36.4)	130(31.5)	26(30.6)	43(37.1)	
Regular exercise					<.001					<.001
No	359(52.6)	396(46.3)	69(42.3)	53(30.3)		184(65.7)	218(52.8)	45(52.9)	40(34.5)	
Yes	309(45.3)	433(50.6)	92(56.4)	119(68.0)		96(34.3)	194(47.0)	40(47.1)	76(65.5)	
Missing	14(2.1)	26(3.0)	2(1.2)	3(1.7)		0(0.0)	1(0.2)	0(0.0)	0(0.0)	
First-degree family history of cancer					0.309					0.283
No	407(59.7)	473(55.3)	92(56.4)	106(60.6)		141(50.4)	231(55.9)	51(60.0)	59(50.9)	
Yes	275(40.3)	379(44.3)	71(43.6)	68(38.9)		139(49.6)	182(44.1)	34(40.0)	57(49.1)	
Missing	0(0.0)	3(0.4)	0(0.0)	1(0.5)		0(0.0)	0(0.0)	0(0.0)	0(0.0)	
First-degree family history of colorectal cancer					0.363					0.402
No	634(93.0)	795(93.0)	151(92.6)	168(96.0)		265(94.6)	379(91.8)	81(95.3)	107(92.2)	
Yes	48(7.0)	57(6.7)	12(7.4)	6(3.4)		15(5.4)	34(8.2)	4(4.7)	9(7.8)	
Missing	0(0.0)	3(0.3)	0(0.0)	1(0.6)		0(0.0)	0(0.0)	0(0.0)	0(0.0)	
Fruit and vegetable intake, g/d^2^	290.4 ± 191.3	325.6 ± 217.1	384.8 ± 223.5	418.6 ± 251.4	<.001	383.1 ± 256.5	424.7 ± 265.5	476.3 ± 218.1	514.2 ± 280.0	<.001
Red meat intake, g/d^2^	60.2 ± 40.5	62.9 ± 40.9	66.1 ± 38.5	56.1 ± 38.4	0.071	39.9 ± 25.0	44.9 ± 28.3	48.5 ± 34.4	37.4 ± 26.8	0.004
Energy intake, kcal/d^2^	1803.6 ± 543.5	1860.2 ± 555.8	1937.0 ± 596.8	2045.0 ± 621.7	<.001	1597.4 ± 522.3	1633.4 ± 549.2	1840.8 ± 605.4	1885.5 ± 642.5	<.001

^a^P-values were calculated by using the chi-square test for categorical variables and linear regression for continues variables. ^b^ Mean (s.d.) ^c^ Fruit and vegetable intake and red meat intake were adjusted for total individual energy intake by using the residual method

Table 3 shows ORs and 95% CIs of colorectal cancer risk according to the frequency of nut consumption. After adjustment for age, education level, alcohol consumption, BMI, regular exercise, red meat intake, fruit and vegetable intake, and total energy intake, a significant inverse relationship was observed between colorectal cancer risk and nut consumption among both men and women (OR: 0.28, 95%CI: 0.17–0.47 for ≥3serving per week vs. none, p for trend = <.001 for men; OR: 0.30, 95%CI: 0.15–0.60 for ≥3serving per week vs. none, p for trend = <.001 for women).

**Table 3:** Odds ratios (OR) and 95% confidence intervals (Cl) for colorectal cancer sub-site in relation to nut consumption

	Control		Colorectal cancer			Proximal colon			Distal colon			Rectum	
Consumption frequency	No	No	Crude OR (95% CI)	Multivariate OR (95% CI)*	No	Crude OR (95% CI)	Multivariate OR (95% CI)*	No	Crude OR (95% CI)	Multivariate OR (95% CI)*	No	Crude OR (95% CI)	Multivariate OR (95% CI)*
All	1846	923			166			291			444		
None	585	377	1.00	1.00	66	1.00	1.00	124	1.00	1.00	179	1.00	1.00
< 1 serving per week	832	436	0.81 (0.68–0.97)	0.91 (0.74–1.12)	73	0.78 (0.55–1.10)	0.67 (0.60–1.25)	135	0.77 (0.59–1.00)	0.85 (0.64–1.14)	218	0.86 (0.68–1.07)	0.96 (0.74–1.24)
1–3 servings per week	185	63	0.53 (0.39–0.72)	0.62 (0.43–0.91)	18	0.86 (0.50–1.49)	0.97 (0.54–1.77)	17	0.43 (0.25–0.74)	0.49 (0.28–0.88)	25	0.44 (0.28–0.69)	0.51 (0.31–0.85)
≥ 3 servings per week	224	47	0.30 (0.21–0.42)	0.30 (0.20–0.45)	9	0.33 (0.16–0.67)	0.32 (0.15–0.69)	15	0.29 (0.17–0.51)	0.29 (0.16–0.53)	22	0.30 (0.19–0.47)	0.30 (0.18–0.50)
*P* for trend			< 0.001	< 0.001		0.003	0.011		< 0.001	< 0.001		< 0.001	< 0.001
Men	1250	625			113			178			320		
None	426	256	1.00	1.00	44	1.00	1.00	79	1.00	1.00	127	1.00	1.00
< 1 serving per week	564	291	0.86 (0.70–1.06)	0.90 (0.70–1.16)	49	0.84 (0.55–1.29)	0.85 (0.55–1.34)	77	0.74 (0.53–1.03)	0.77 (0.54–1.11)	160	0.95 (0.73–1.24)	1.00 (0.74–1.36)
1–3 servings per week	116	47	0.67 (0.46–0.98)	0.78 (0.50–1.23)	15	1.25 (0.67–2.33)	1.27 (0.65–2.51)	10	0.47 (0.23–0.93)	0.54 (0.26–1.14)	20	0.58 (0.35–0.97)	0.66 (0.37–1.19)
≥ 3 servings per week	144	31	0.36 (0.24–0.54)	0.28 (0.17–0.47)	5	0.34 (0.13–0.86)	0.25 (0.09–0.70)	12	0.45 (0.24–0.85)	0.39 (0.19–0.80)	13	0.30 (0.16–0.55)	0.23 (0.12–0.46)
*P* for trend			< 0.001	< 0.001		0.098	0.050		0.001	0.004		< 0.001	< 0.001
Women	596	298			53			113			124		
None	159	121	1.00	1.00	22	1.00	1.00	45	1.00	1.00	52	1.00	1.00
< 1 serving per week	268	145	0.71 (0.52–0.97)	0.94 (0.65–1.38)	24	0.65 (0.35–1.19)	0.99 (0.50–1.94)	58	0.77 (0.49–1.18)	0.99 (0.61–1.63)	58	0.66 (0.43–1.01)	0.87 (0.54–1.40)
1–3 servings per week	69	16	0.31 (0.17–0.55)	0.37 (0.18–0.77)	3	0.31 (0.09–1.09)	0.51 (0.13–2.00)	7	0.36 (0.15–0.83)	0.39 (0.15–1.03)	5	0.22 (0.09–0.58)	0.26 (0.10–0.76)
≥ 3 servings per week	100	16	0.21 (0.12–0.38)	0.30 (0.15–0.60)	4	0.29 (0.10–0.86)	0.54 (0.17–1.83)	3	0.11 (0.03–0.35)	0.13 (0.04–0.48)	9	0.28 (0.13–0.58)	0.40 (0.17–0.95)
*P* for trend			< 0.001	< 0.001		0.007	0.222		< 0.001	< 0.001		< 0.001	0.006

Adjusted for age(< 50 years, 50–59, 60 or older), education(less than high school, high school, college or above, and missing) alcohol consumption(non-drinker, ex-drinker, current drinker, and missing), regular exercise(no, yes, and missing), body mass index(< 25 kg/m^2^, ≥25 kg/m^2^, and missing), red meat(continuous), fruit and vegetable intake (continuous), calcium(continuous), vitamin D(continuous), and total energy intake(continuous)

In sub-site analyses, among men who consumed ≥3 servings per week, a reduction in risk was observed for proximal colon cancer (OR: 0.25, 95% CI: 0.09–0.70), distal colon cancer (OR: 0.39, 95% CI: 0.19–0.80), and rectal cancer (OR: 0.23 95% CI: 0.12–0.46) compared to men who consumed none. Similarly, compared to women who did not consume nuts, women who consumed ≥3 servings per week showed an inverse association between nut consumption and risk of colorectal cancer overall, as well as distal colon cancer (OR: 0.13 95% CI: 0.04–0.48) and rectal cancer (OR: 0.40 95% CI: 0.17–0.95).

## Discussions

Our case-control study suggests a favorable association between high frequency of nut consumption and decreased risk of colorectal cancer among both men and women. The results of sub-site analyses showed an inverse association with all sub-sites of colorectal cancer, except for proximal colon cancer for women.

Our results are consistent with some previous studies. Findings from a meta-analysis of 3 cohort studies found a 24% decrease in the risk of colorectal cancer for the highest category of nut consumption [13]. A cohort study of women in Taiwan found that frequent peanut intake was associated with an approximately 58% reduction in the risk of colorectal cancer [23]. The European Prospective Investigation into Cancer and Nutrition study observed a null association between consumption of nuts and seeds and the risk of colorectal cancer among women. However, an inverse association was supported for colon cancer (HR: 0.69, 95% CI: 0.50–0.95 for consumption of 6.2 g/day vs. ≥0 g/day) [22]. In previous case-control studies conducted in the US [26] and Korea [30], there were no statistically significant associations between consumption of nuts and other legumes and risk of colorectal cancer. In sub-site analyses, the preventive effect was observed only for cancer in the distal colon and rectum. One of two existing studies found no statistically significant results regardless of anatomical site [20], and the other study found differences according to sub-site [22]. Clinical and molecular characteristics are different according to the anatomical location of colorectal cancer [31]. The relationship of some dietary ingredients with distal colon and rectal cancer risk was stronger than with proximal colon cancer [32]. There is a general lack of studies on the relationship between nut intake and colorectal cancer, so no clear cause for these differences is known.

Despite the similarity of our results with previous literature, there were differences in the assessment of nut consumption. First, our serving size (1 serving size = 15 g) was smaller than in studies conducted in other countries (1 serving size = 28 g). Second, in our study the only types of nuts included in the SQFFQ were pine nuts, peanuts and almonds, which may be difficult to compare with studies that included seeds, legumes, peanut butter and country-specific nuts. Third, the percentage of people who have allergies to nuts varies from country to country [33]. Previous studies have shown that people born in Asia have a relatively low risk for peanut and tree nut allergies compared to those born in Western countries [34, 35].

To explain the association between nut consumption and colorectal cancer risk, several biological pathways have been proposed. Peanuts are known to be rich in isoflavones, phytosterols, resveratrol and phenolic acid [36–38], which may have anti-cancer effects. Phytosterols, especially beta-sitosterol, have been shown both in vivo and in vitro to help normalize hyper-proliferating cells and to inhibit colon cancer [38, 39]. Resveratrol is a natural polyphenolic antioxidant, which decreases cyclins D1 and D2 and regulates the cell cycle [40]. Almonds and pine nuts contain fiber, resveratrol, selenium, flavonoids (quercetin), polyphenols (ellagic acid), and folic acid, which may prevent cancer through antioxidants, regulation of cell differentiation and proliferation, reduction of DNA damage, regulation of inflammatory response and immunological activity [17]. Each type of nut has many nutrients and phytochemicals that may be beneficial to health, and it is likely that unknown harmony effects of nuts may be related to colorectal cancer prevention. Moreover, many studies have shown a beneficial association between high nut intake and decreased risk of obesity [41–43] and type 2 diabetes [44, 45], which are risk factors for colorectal cancer.

This case-control study is the first exploration of the association between nut consumption and risk of colorectal cancer in the Korean population, where nut consumption frequency and patterns may differ compared with other countries. A number of potential limitations may influence the present study. First, we did not consider the manufacturing method (raw, roasted, or boiled) or any extra content (sugar, salt, seasoning, etc.). In Korea, most nuts are consumed in a processed form or as a garnish. Different preparation methods before and after cooking, time, and temperature conditions can affect nutrient composition and content of nuts [46, 47]. Second, we could not eliminate the possibility of some residual and unmeasured confounding. Previous studies have reported that people who consume nuts were more likely to have other healthier behaviors, such as lower intakes of alcohol and sodium, lower BMI, higher physical activity levels, better dietary quality, and higher socioeconomic status [9, 10, 48–51]. Our study had similar findings in terms of higher education, more regular exercise, and higher intakes of energy and fruits and vegetables. Thus, we adjusted for major potential confounders in our analyses. Third, the item ‘nuts’ in the SQFFQ used in this study cannot represent all nuts, because it only included peanuts, pine nuts, and almonds. For this reason, direct comparisons with the results of other studies may be difficult. Fourth, hospital controls who voluntarily participate in studies such as ours may be more likely to have a healthier lifestyle compared with cancer patients. For comparability, we recruited cases and controls in the same hospital, and adjusted for factors that were different between groups. Fifth, because of the case-control study design, recall bias is an inherent weakness. It is known that people who are conscious of health may over-report or under-report some food items [52]. However, the protective effect of nuts on colorectal cancer was not generally known at the time of the survey and thus should be unrelated to recall bias. Finally, due to the small number of participants in the highest nut consumption category, we cannot rule out the possibility that some of our results are due to chance. However, the associations were consistent in both the sub-site analyses and analyses stratified by sex, which reduces the likelihood of chance findings.

Our study had a retrospective design, which is regarded to have a lower value in terms of evidence hierarchy compared with prospective studies. Nevertheless, case-control studies can provide evidence supporting the general relationship between diet and cancer, while currently available prospective studies lack sufficient duration of follow-up for cancer occurrence. Our results do need to be confirmed in a large cohort study with an appropriate dietary assessment of nut consumption.

## Conclusions

In conclusion, high frequency of nut consumption appears to play a role in decreasing colorectal cancer risk in this study of a Korean population. Our findings could be used to advise the general public about nut consumption.

## Abbreviations

BMI: Body mass index
CI: Confidence intervals
OR: Odds ratios
SQFFQ: Semi-quantitative food frequency questionnaire
